# Supplementary material for: Sex-specific proximal tubular cell differentiation pathways identified by single-nucleus RNA sequencing
Source: Sci Rep. 2024 Oct 14;14:24041. doi: 10.1038/s41598-024-73102-7 (PMC11473948; doi:10.1038/s41598-024-73102-7)

Supp Fig. 1 Data processing and batch effect correction. The violin plots show the number of genes, UMIs and percentage of mitochondrial genes before (A) and after (B) QC step. The dot plots show the result of PCA before (C) and after (D) harmony batch effect correction. The effect of batch effect correction on PC_1 are shown using violin plots: (E) before harmony correction and (F) after harmony correction.


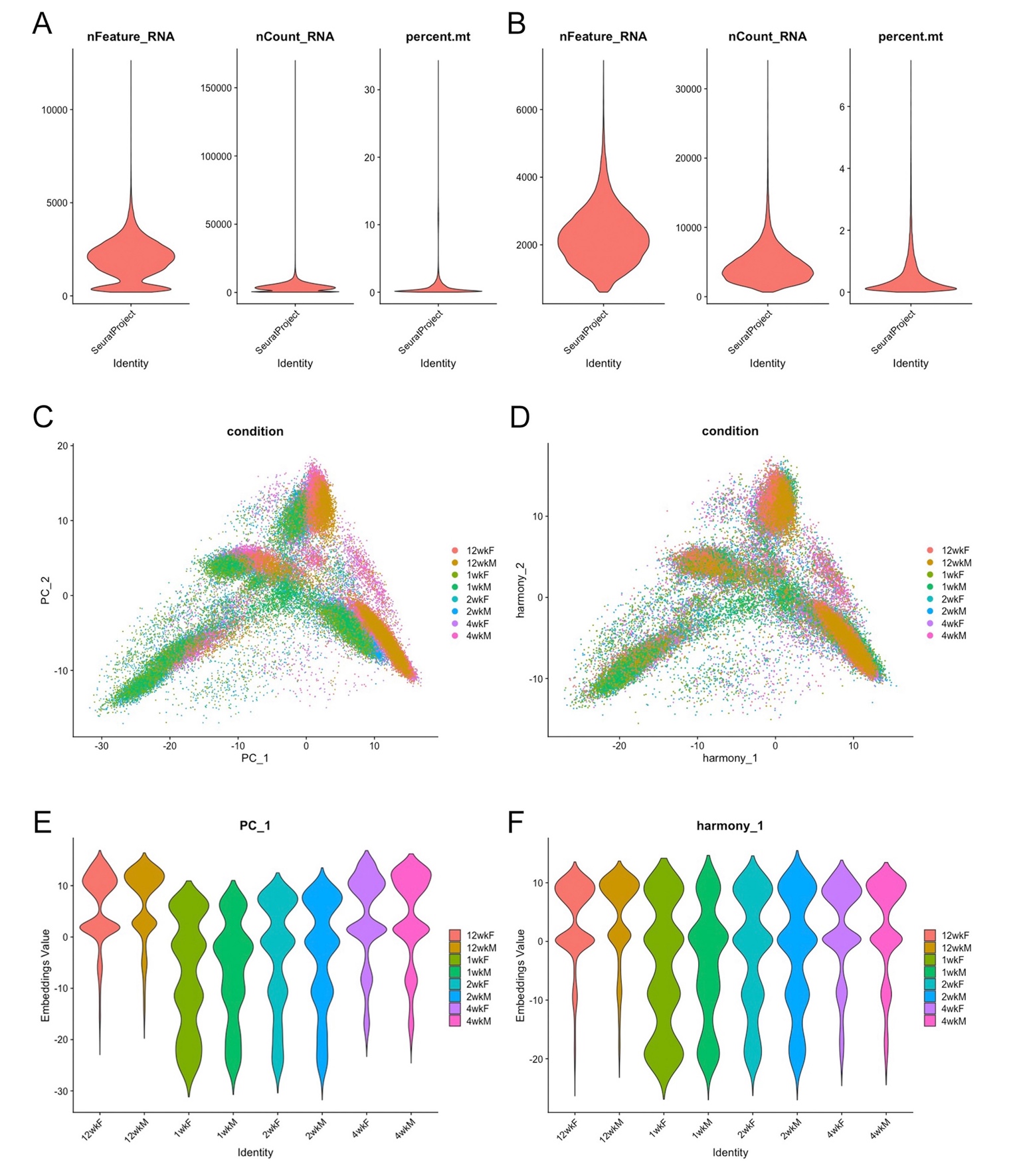


Supp Fig. 2 Result of PTC re-clustering. (A) UMAP plot shows the PTCs are discriminated into 26 clusters; (B) Results of re-cluster are compatible with the primary result of cell type identification; (C) Violin plots show the number of genes detected in each cluster. The proliferative clusters and cluster 25 contain greater numbers of genes per cell; (D, E) Feature plots of the expression of *Flt* and *Emcn* genes.


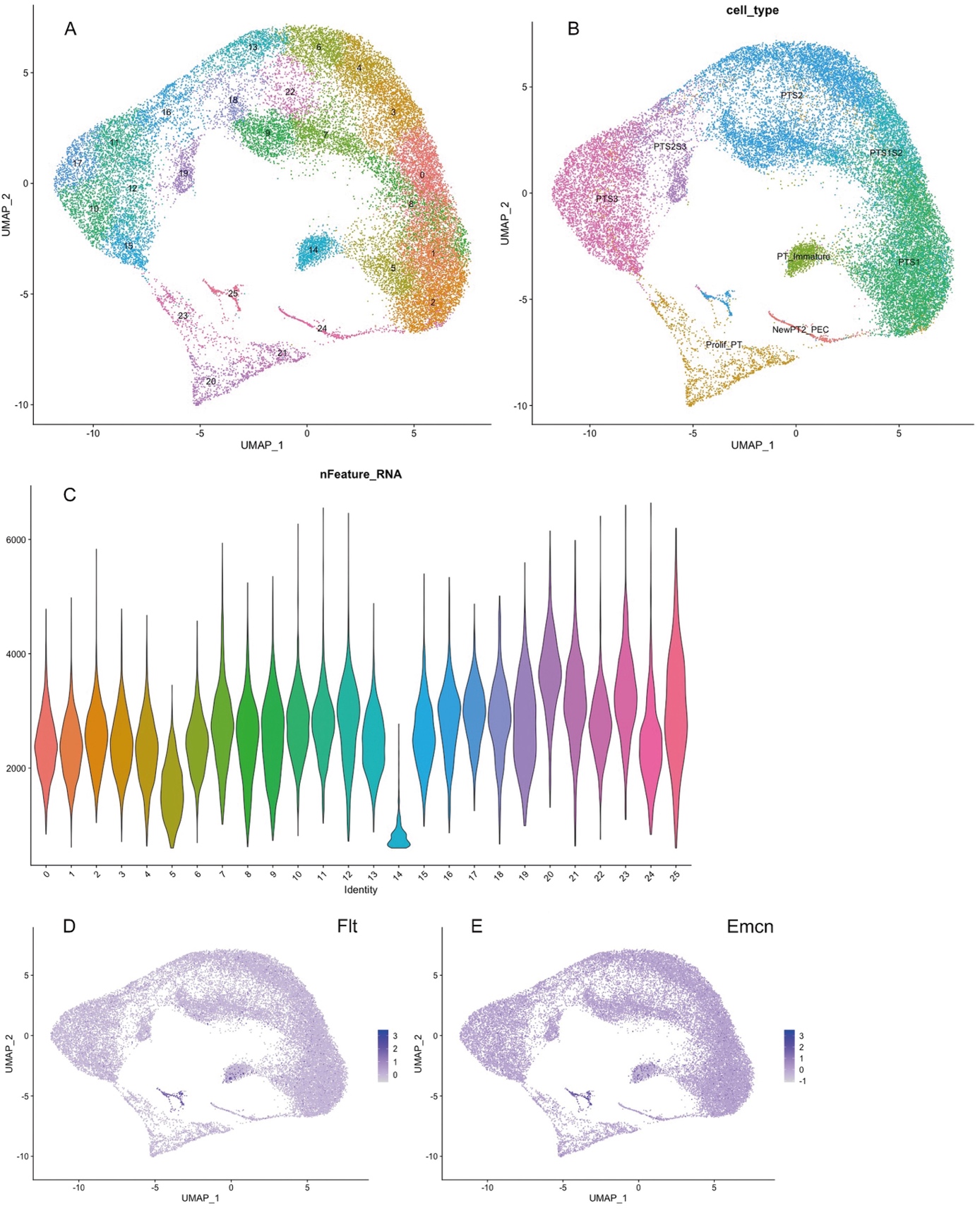


Supp Fig. 3 SnRNA-seq of 69775 nuclei from female and male mouse kidneys at 1, 2 ,4 and 12 weeks of age. Results of cell clustering and cell type identification are shown as uniform manifold approximation and projection (UMAP) plots at different ages.


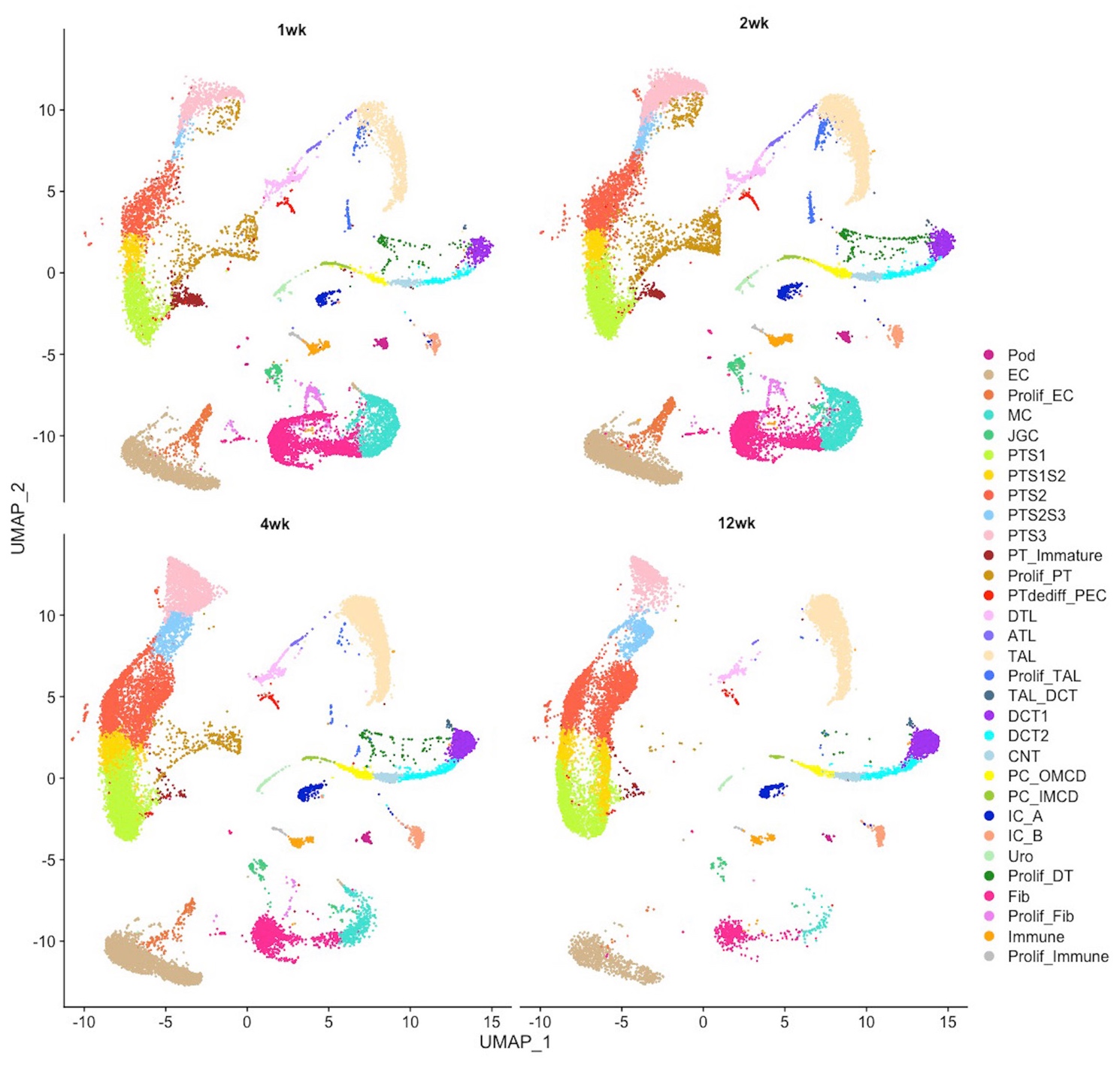


Supp Fig. 4 SnRNA-seq of 69775 nuclei from female and male mouse kidneys at 1, 2 ,4 and 12 weeks of age. Results of cell clustering and cell type identification are shown as uniform manifold approximation and projection (UMAP) plots at different ages and split by female (F) and male (M).


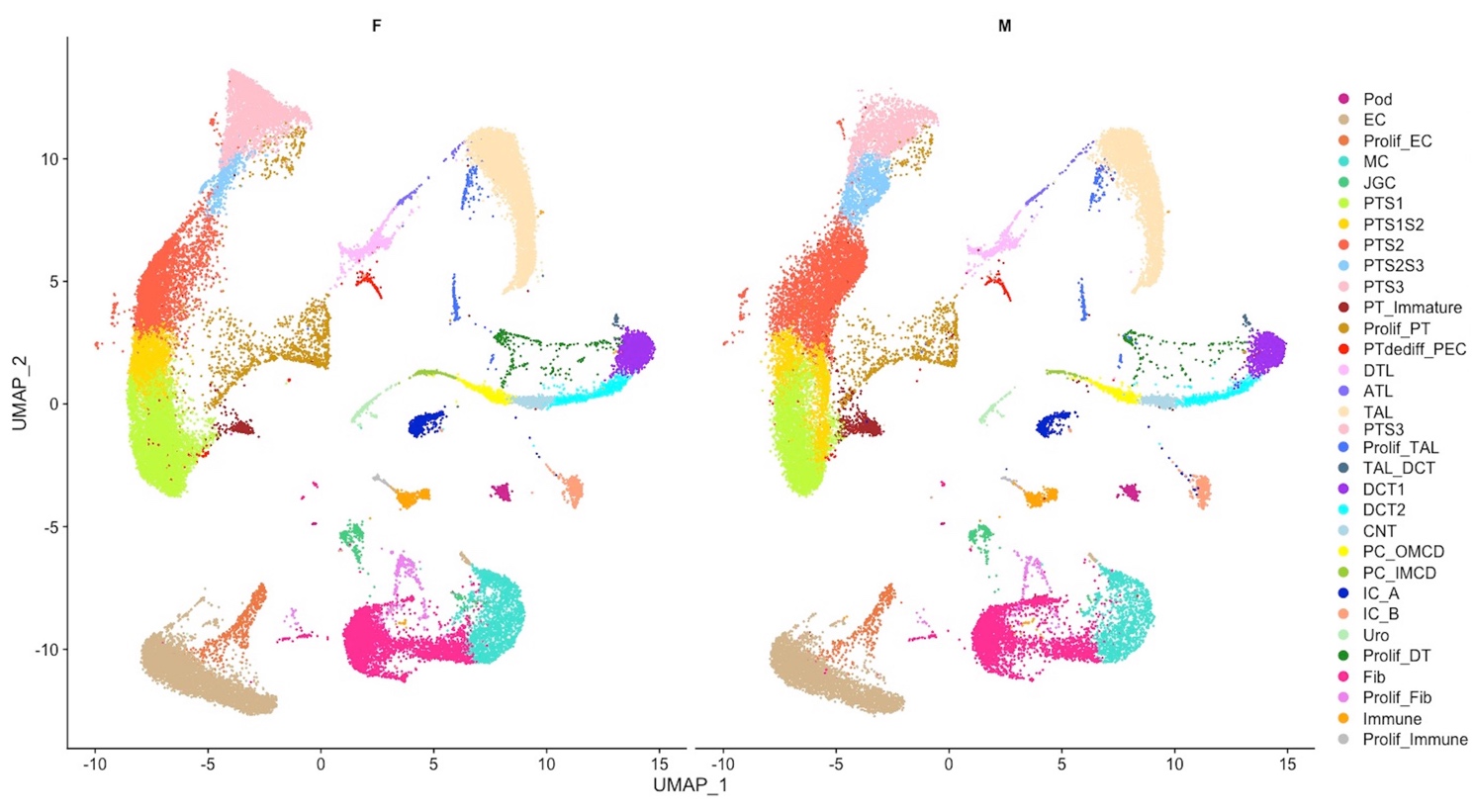


Supp Fig. 5 SnRNA-seq of 69775 nuclei from female and male mouse kidneys at 1, 2 ,4 and 12 weeks of age. Results of cell clustering and cell type identification are shown as female (F) and male (M) uniform manifold approximation and projection (UMAP) plots.


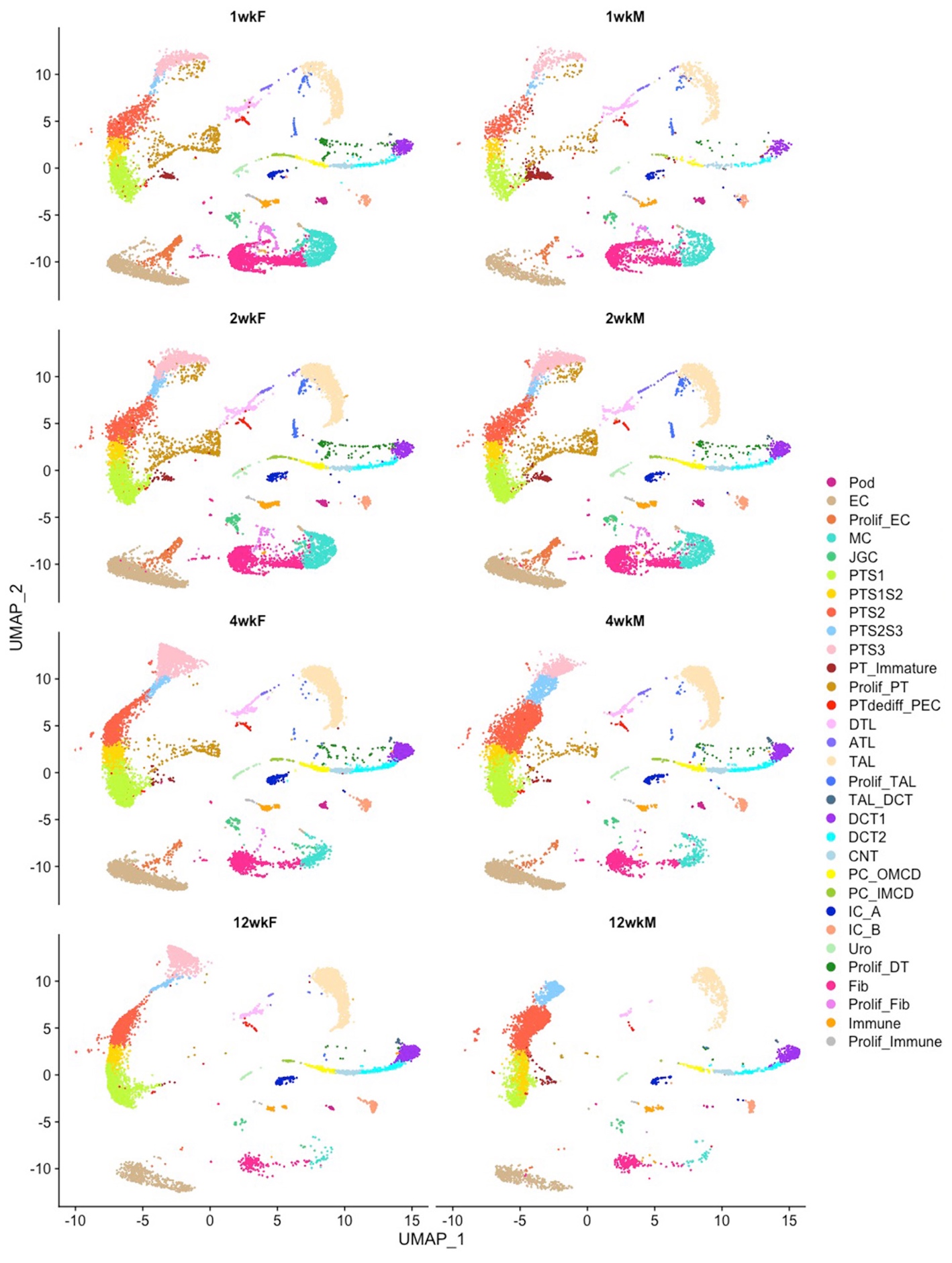


Supp Fig. 6 Line chart of the percentage of each cell type at different ages.

Pod, podocytes; EC, endothelial cells; Prolif_EC, proliferative endothelial cells; MC, mesangial cells; JGC, Juxtaglomerular cells; PT, proximal tubular cells; S1/S2/S3, segment 1/2/3 of proximal tubule; Prolif_PT, proliferative proximal tubular cells; PTdediff_PTC, dedifferentiated proximal tubular_parietal cells; DTL, descending thin limb cells; ATL, ascending thing limb cells; TAL, thick ascending limb cells; Prolif_TAL, proliferative thick ascending limb cells; TAL_DCT, thick ascending limb_ distal convoluted tubule cells; DCT1/DCT2, distal convoluted tubular ½ cells; CNT, connecting tubular cells; PC_OMCD, principal cell-outer medullary collecting duct cells; PC_IMCD, principal cell-inner medullary collecting duct cells; IC_A, intercalated cells, type A; IC_B, intercalated cells, type B; Fib, fibroblasts; Prolif_Fib, proliferative fibroblasts; Uro, urothelial cells.


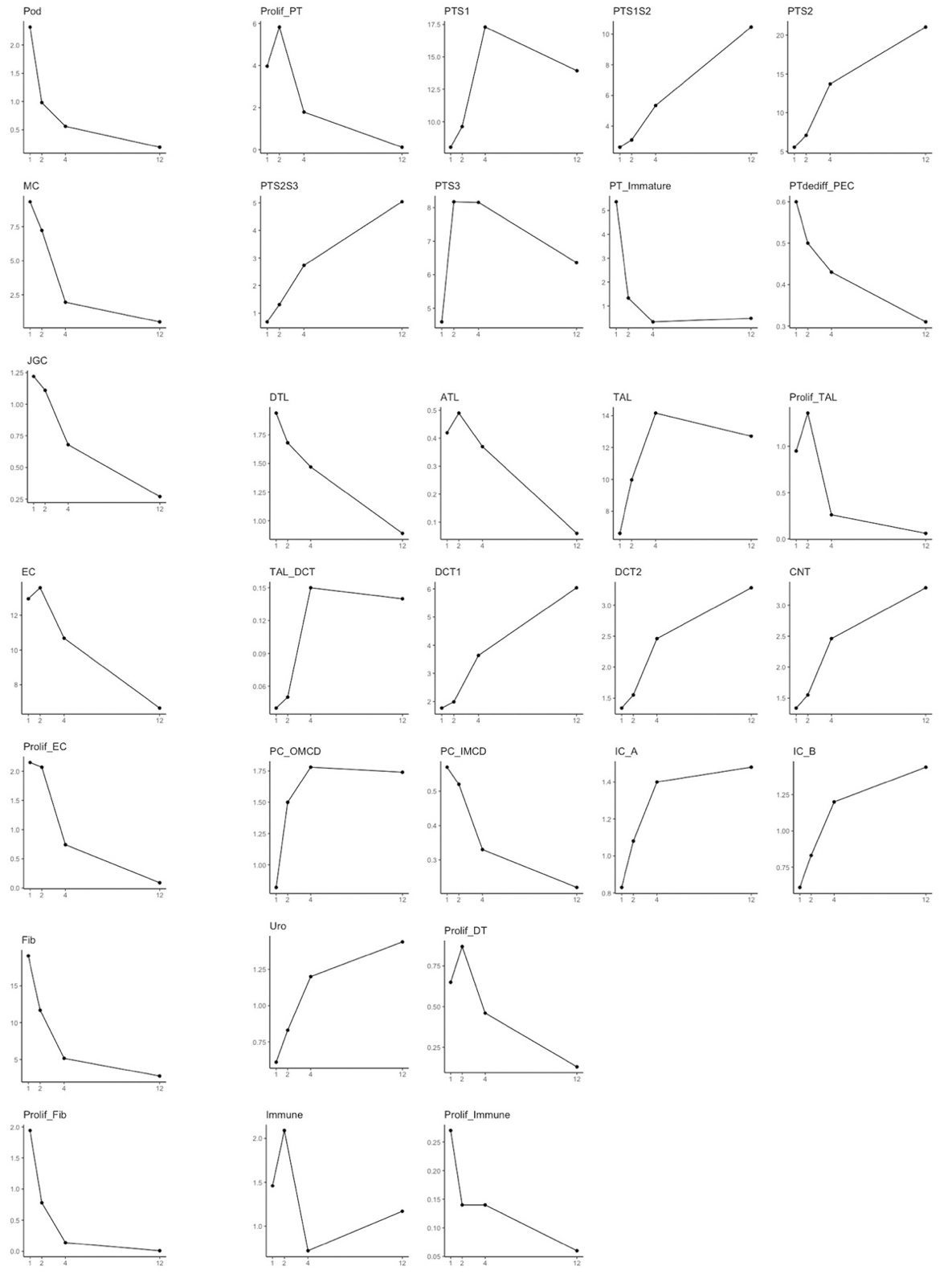


Supp Fig. 7 UMAP plots of re-clustered PTCs at different ages.


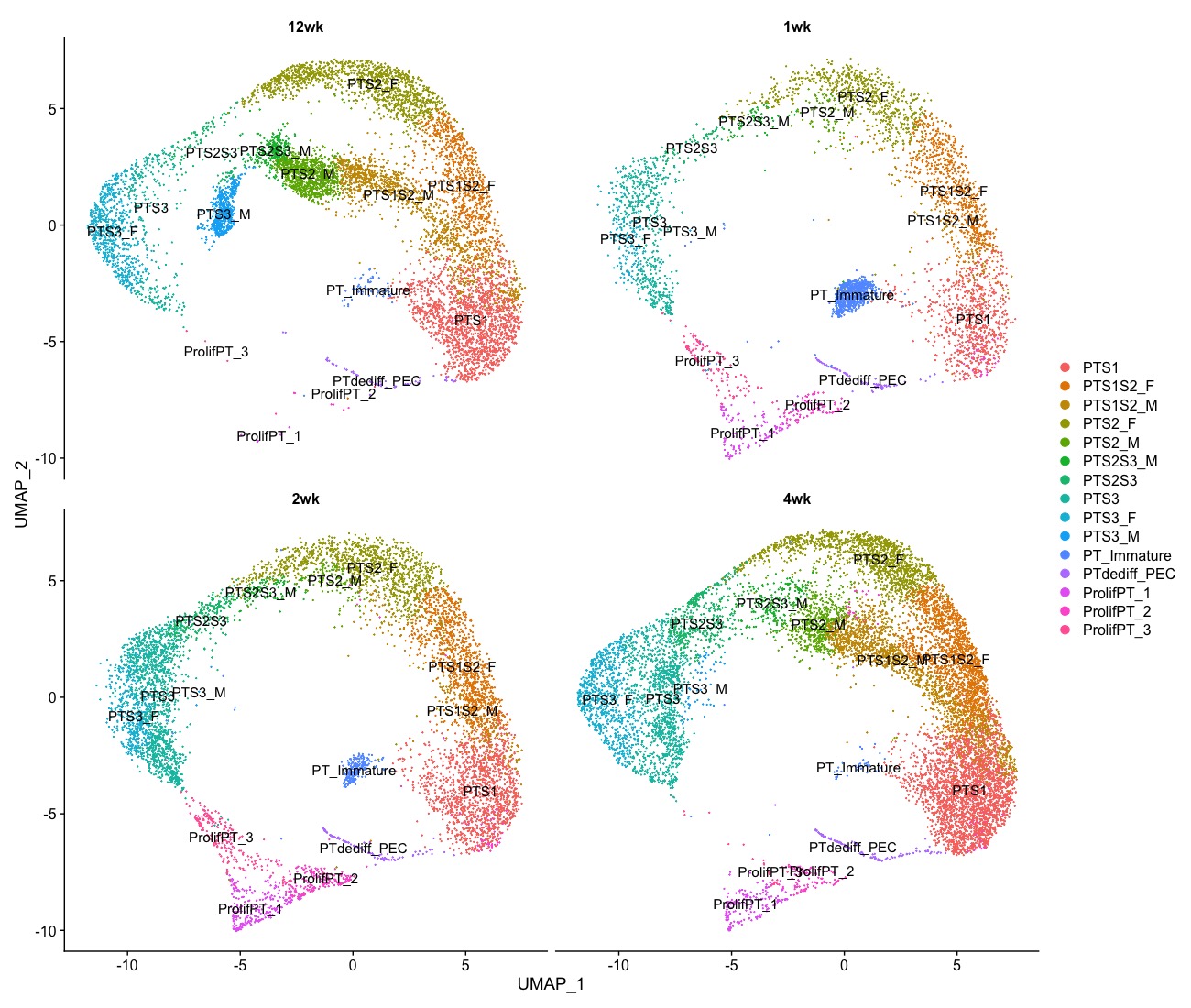


Supp Fig. 8 Female (F) and male (M) UMAP plots of re-clustered PTCs


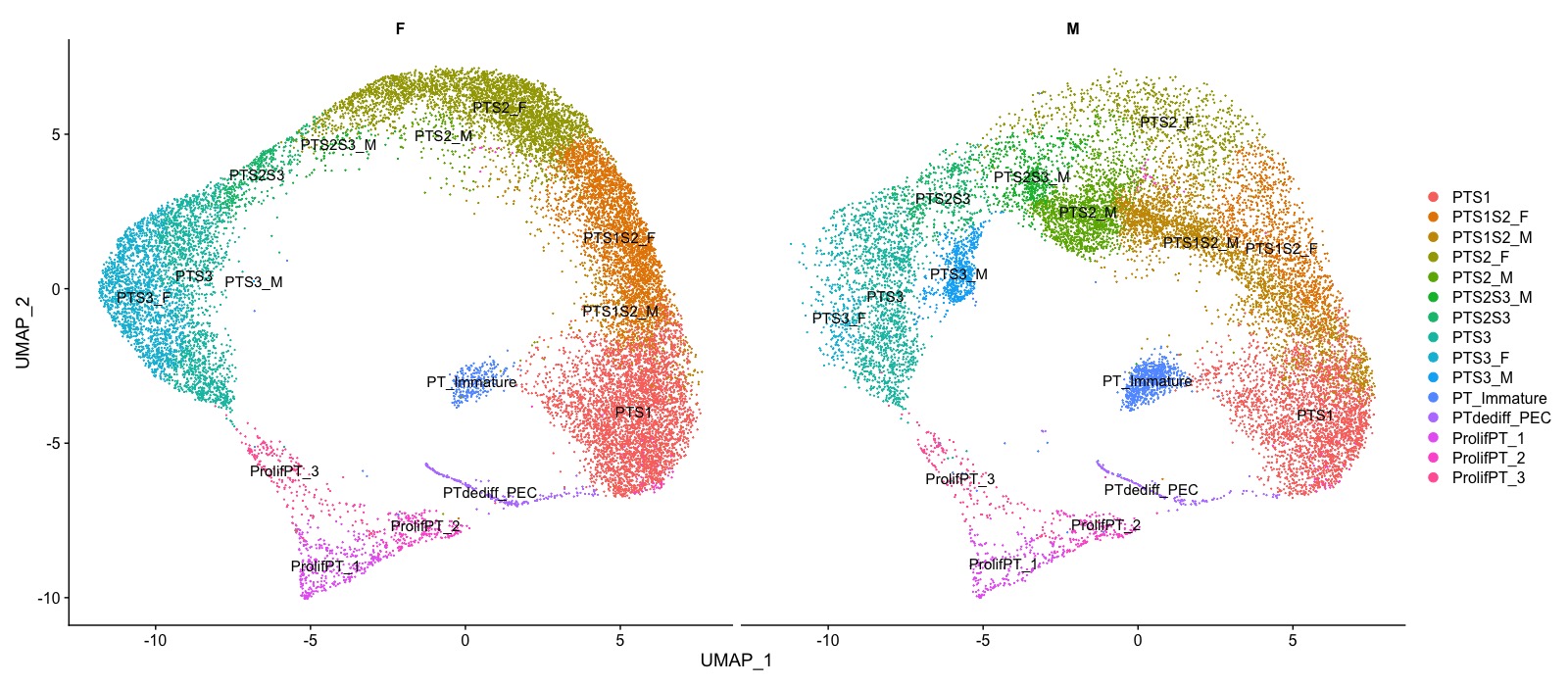


Supp Fig. 9 Dotplot shows the expression levels and the percentage of gene expression of PTC marker genes of female (F, red dots) and male (M, blue dots) cells. *Xist* expressed on female clusters and *Uty* expressed on male clusters.


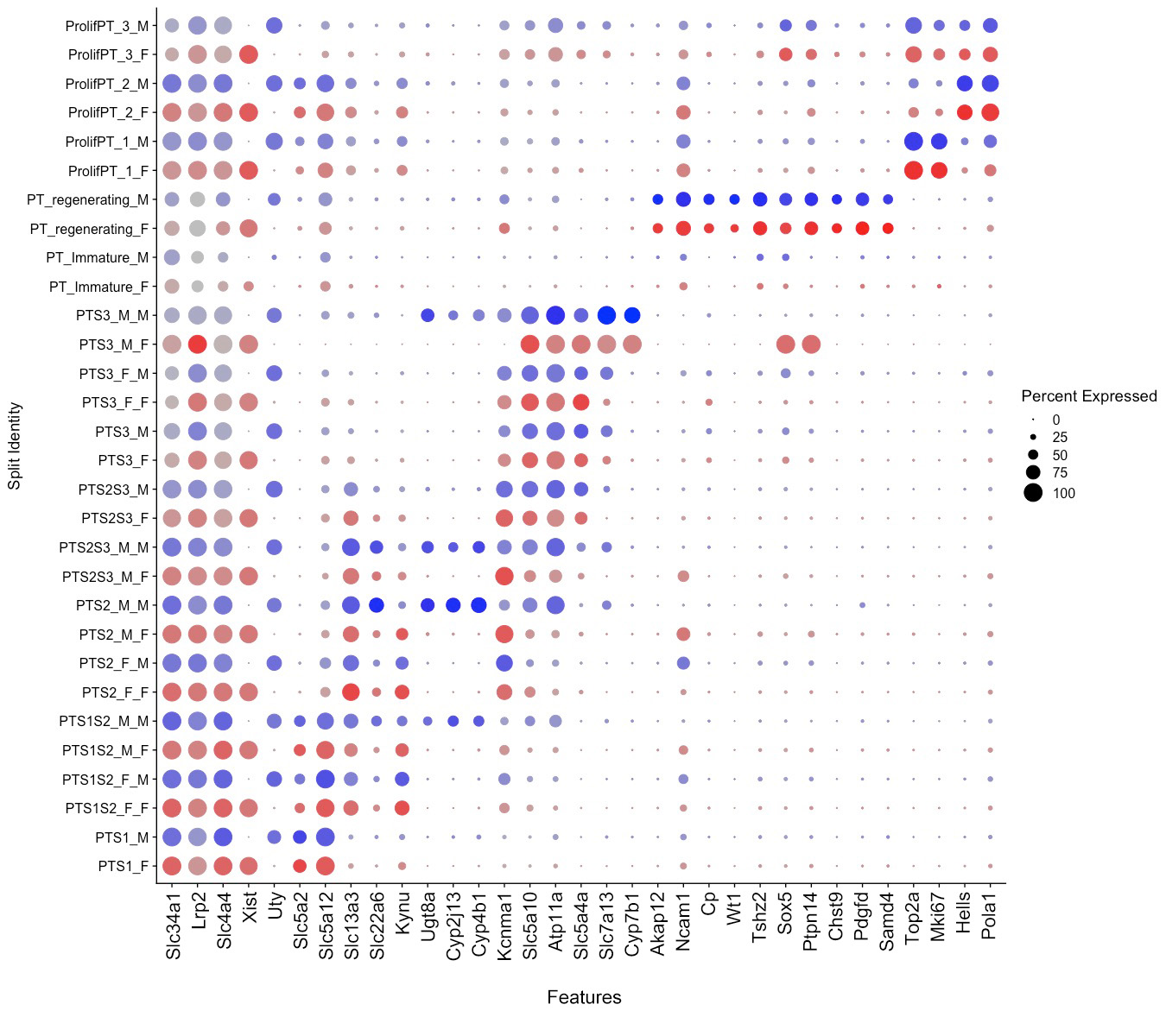


Supp Fig. 10 Dotplot shows the expression levels and the percentage of gene expression of PTC marker genes in all types of cells.


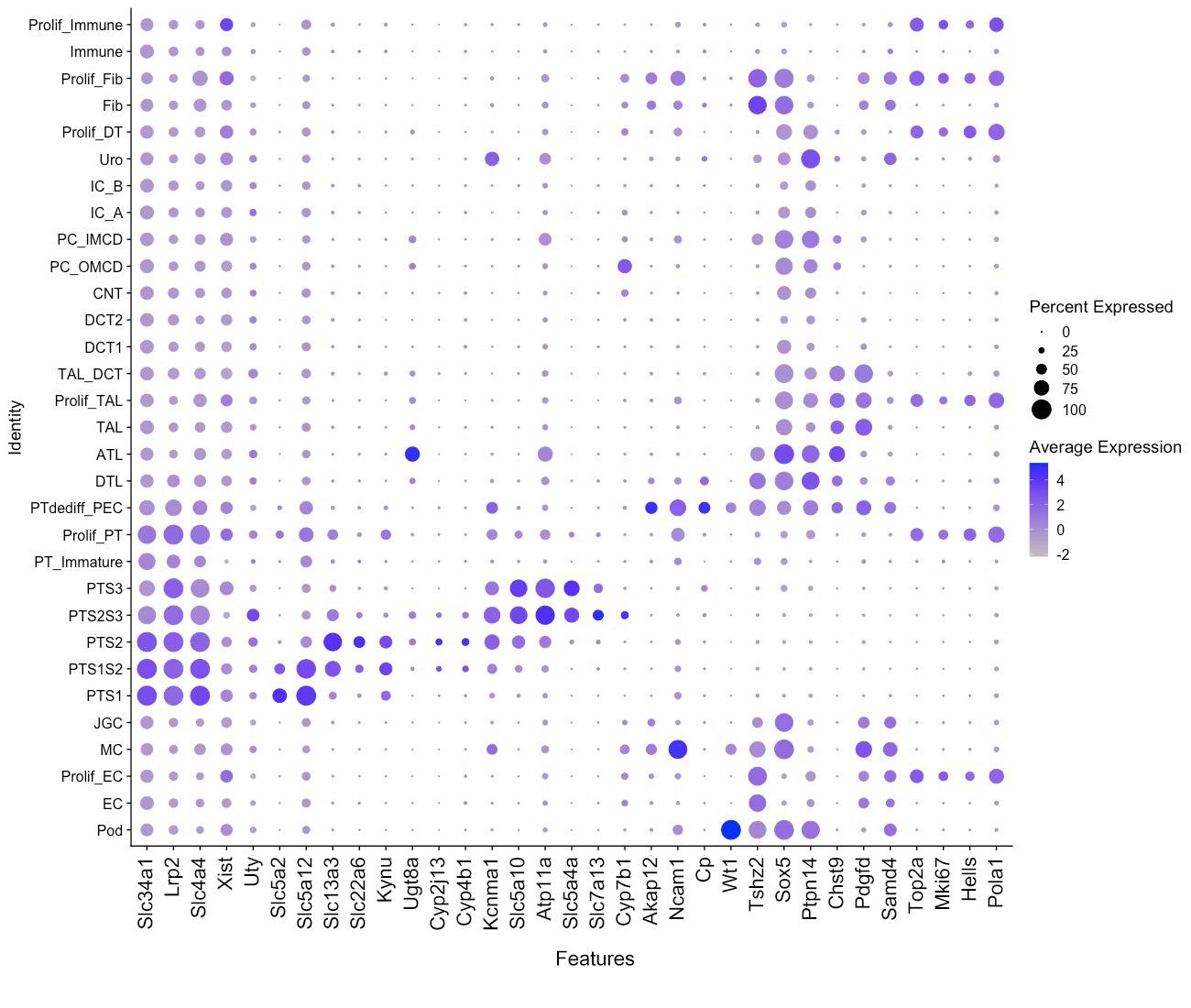


Supp Fig. 11 Trajectory analyses with alternate starting points. Individual trajectories have been generated for origins including each of the individual proliferating clusters ProlifPT1, ProlifPT2 and ProlifPT3, and for the combined proliferating clusters plus immature PT (ProlifPT+Immature). Trajectories and cluster/nuclei orientation along the trajectory are similar in all cases, and similar to that presented in the main manuscript (Fig. 5B) which has the origin of the three proliferating PT clusters.


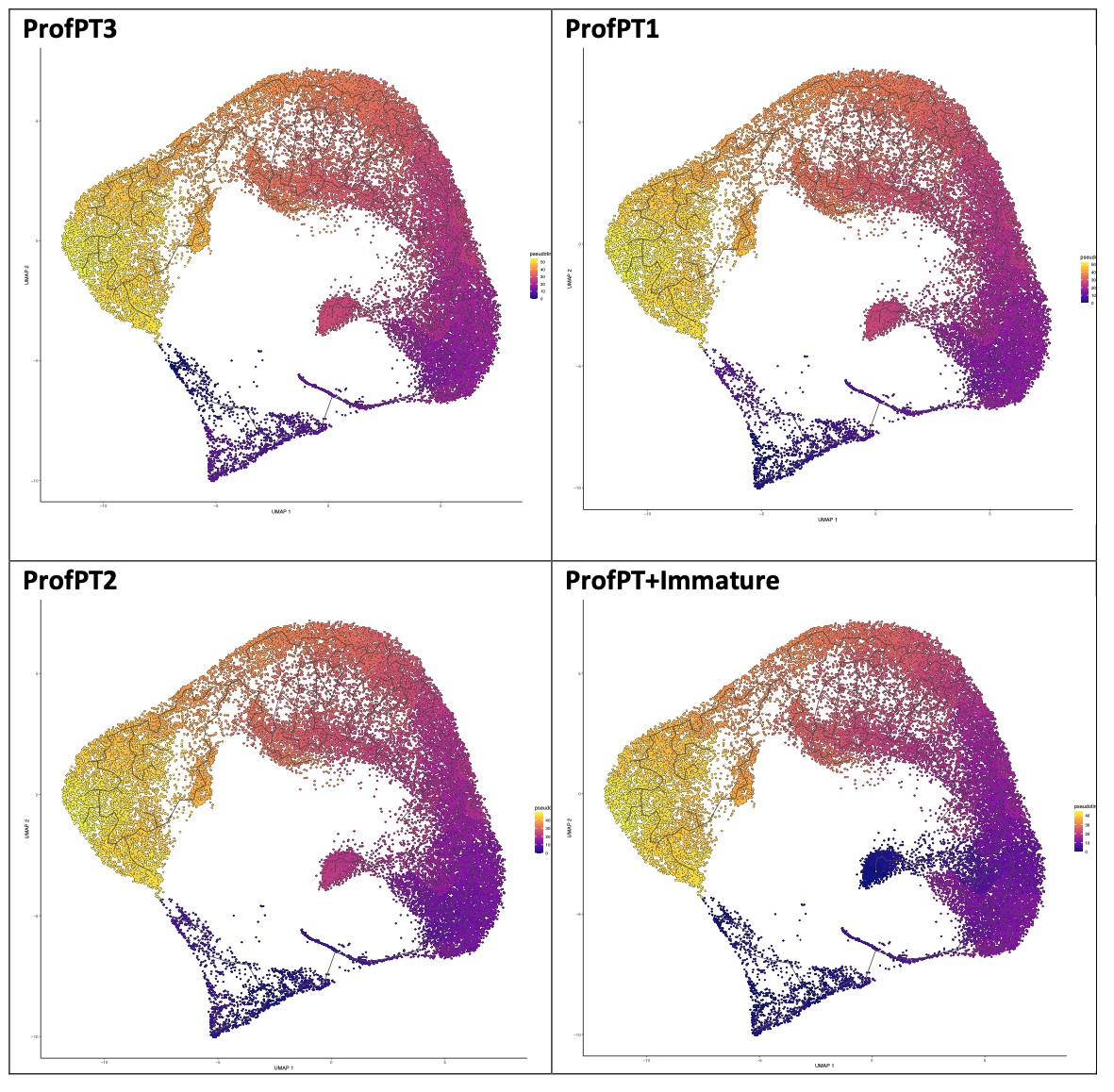


Suppl. Fig 12. Gene expression along pseudotime for the presented trajectories, calculated separately for origins of each of the individual proliferating clusters ProlifPT1, ProlifPT2 and ProlifPT3, and for the combined proliferating clusters plus immature PT (ProlifPT+Immature). Gene expression along pseudotime is presented for marker genes of the PTC clusters (blue trend lines) and for genes selected from DEG analysis as differentially expression in male versus female kidney, in male (green trend lines) and female (red trend lines) mouse kidneys. *Xist, Uty, Ddx3y and Kdm5d* were persistently expressed throughout PTC differentiation whereas other genes demonstrate restricted expression patterns. Results are similar in all cases to those presented for the core analysis which had the specified origin of all ProlifPT clusters (Fig 5 C, D).


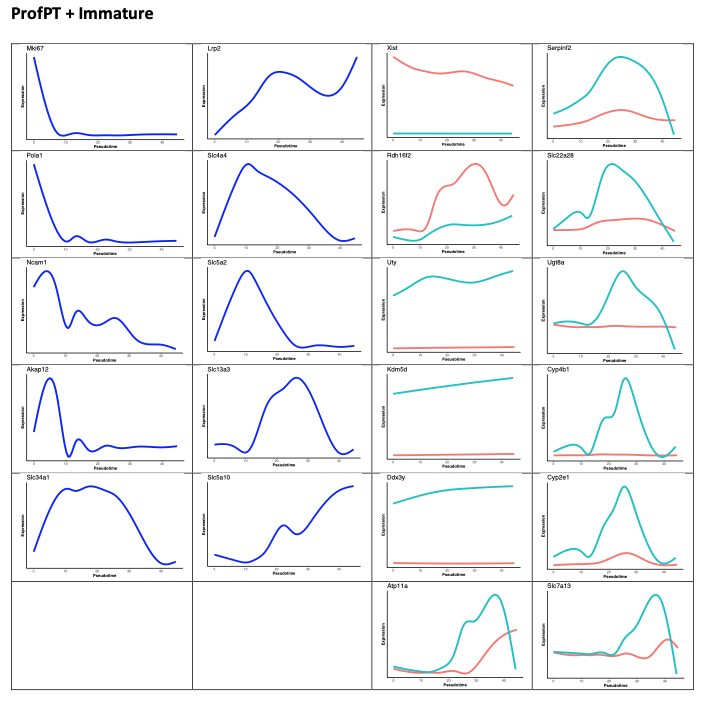


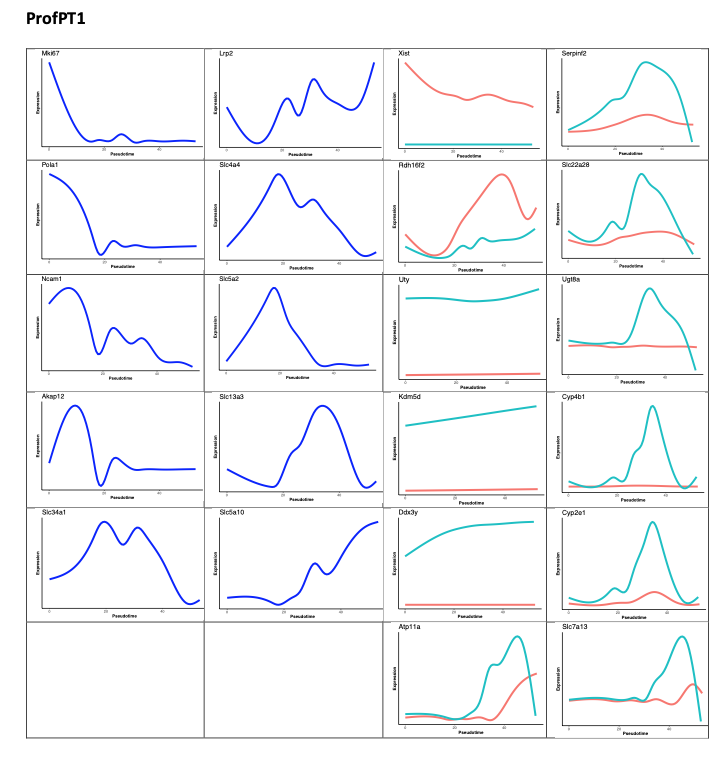


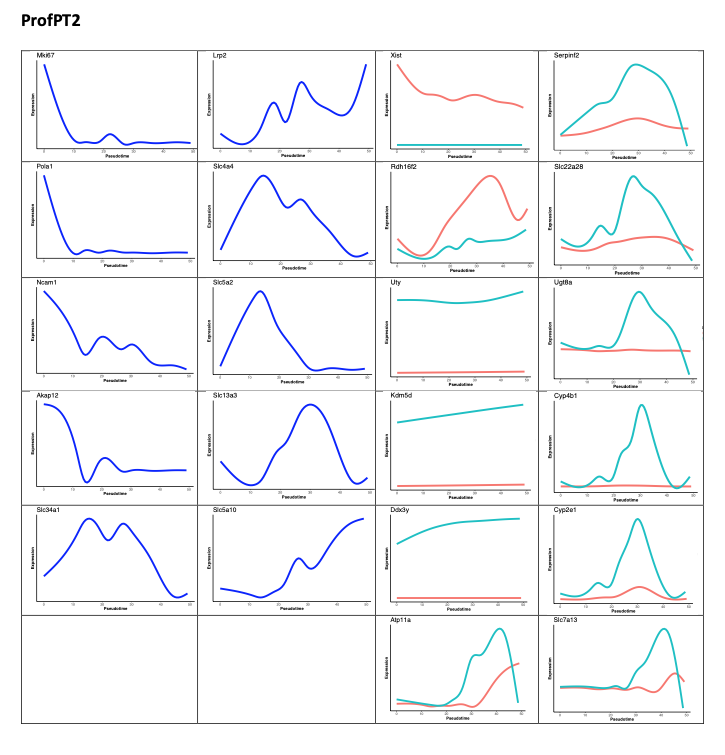


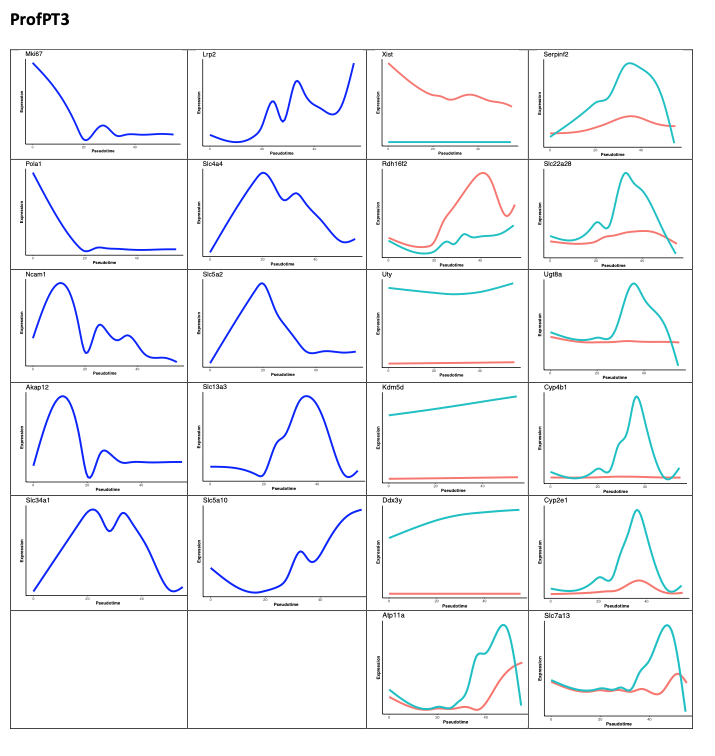

Supplement: Supplementary file 2 — Supplementary Material 2 [file 41598_2024_73102_MOESM2_ESM.docx]
